# Supplementary material for: Acute Myeloid Leukemia Expresses a Specific Group of Olfactory Receptors
Source: Cancers (Basel). 2023 Jun 6;15(12):3073. doi: 10.3390/cancers15123073 (PMC10296317; doi:10.3390/cancers15123073)
Supplement: Supplementary file 1 [file cancers-15-03073-s001.zip › Supplementary Figures.pdf]

Supplementary Information

Supplementary Figures S1-S6

A)

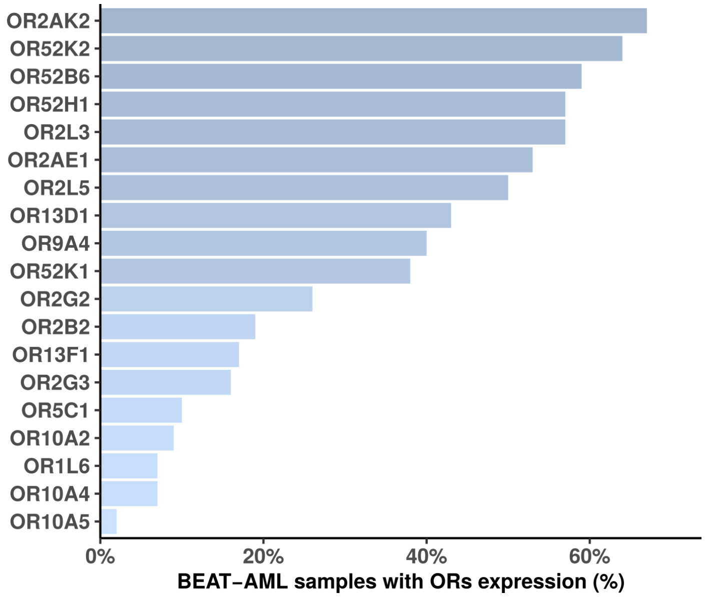

B)

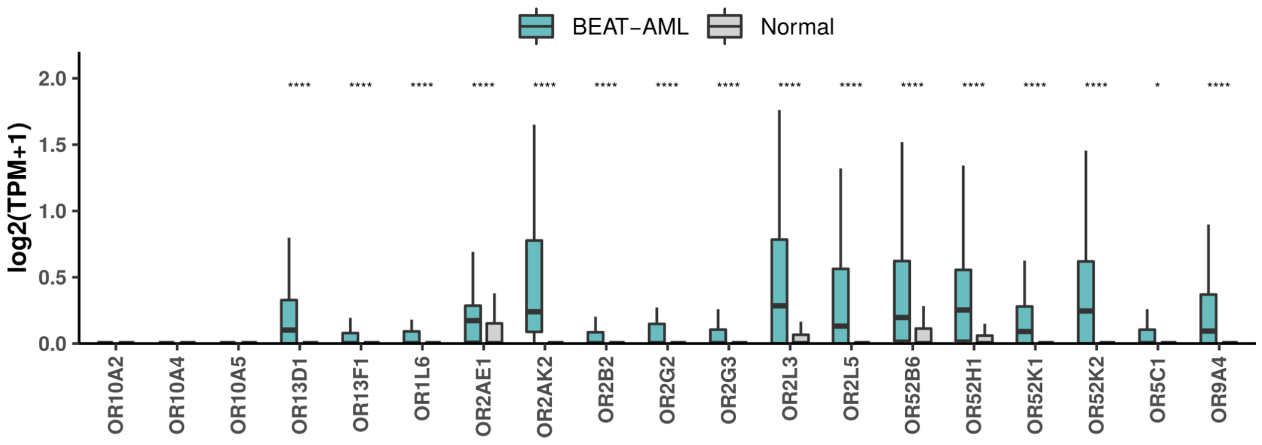

Figure S1. Expression of 19 ORs in samples from the BEAT - AML consortium. A) Percentage of AML samples showing expression of ORs. B) Boxplot showing expression of 19 ORs in AML or Normal samples. "*": p-value < 0.01; "****": p-value < 0.00001;

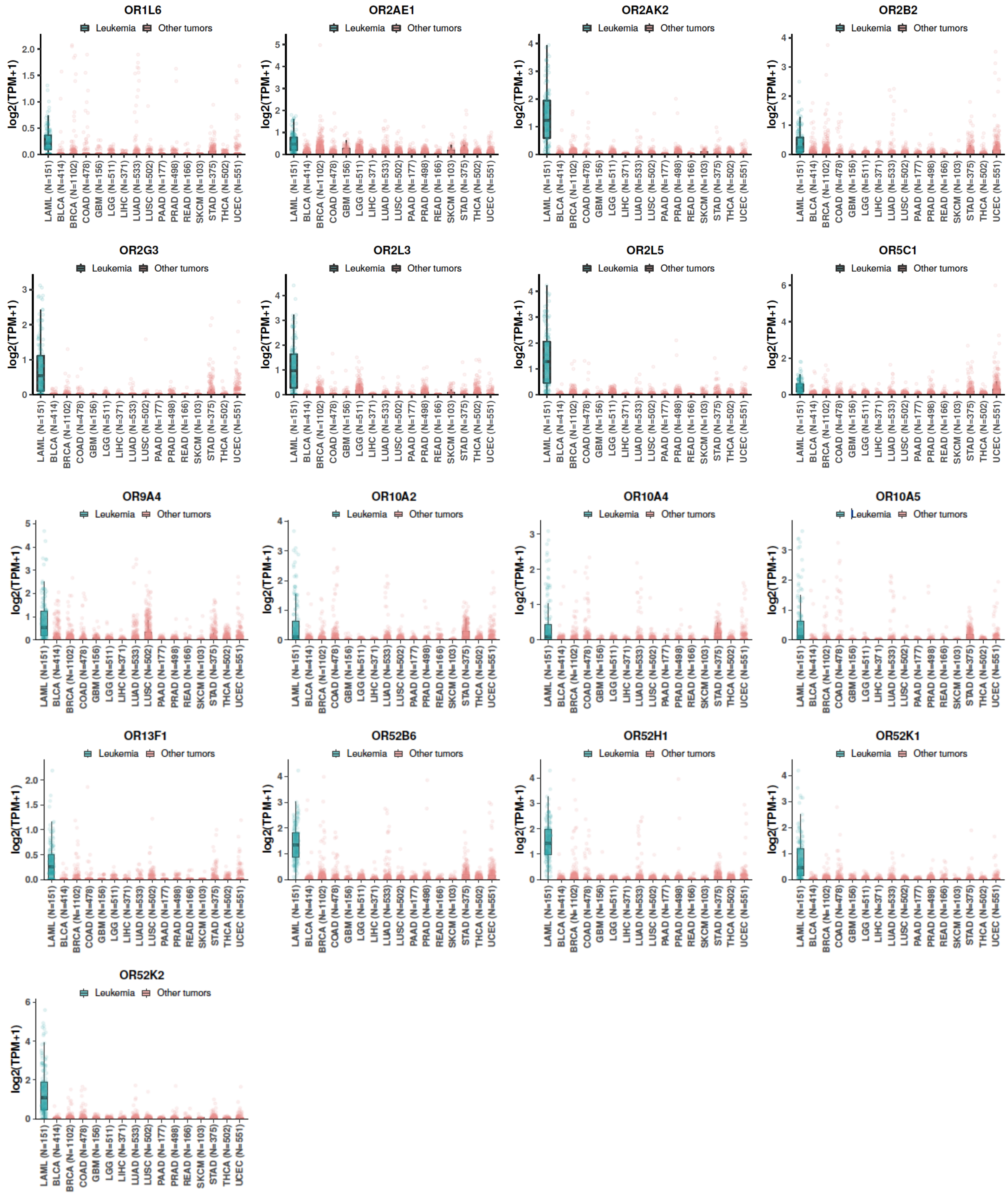

Figure S2. Expression of 19 individual ORs in AML and 15 other cancers from the TCGA. Expression unit is shown in log2(TPM +1). Only tumors with RNAseq data were used.

<table>
<tr><th>ID</th><th>Alignment of Ref Seq and Sample Electropherogram</th><th>BLAST Alignment</th></tr>
<tr><td>OR13F1 forw</td><td>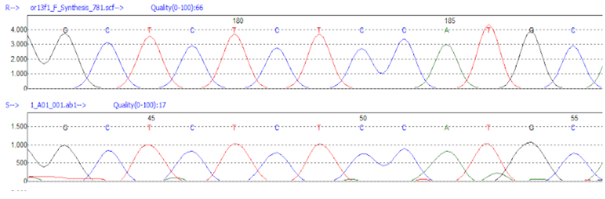
</td><td>Score 139 bits(75) · Expect 2e-30 · Identities 93/101(92%) · Gaps 3/101(2%) · Strand Plus/Plus
Features: olfactory receptor 13f1
Query 10 CCACCTGATGGTGGTAG-TTTGTTCTATGGGATGGCTCTCTCCATGCACCTGAAGCCTTC 68
Sbjct 116679777 CCACCTGATGGTGGTAGTTTTGTTCTATGGGATGGCTCTCTCCATGCACCTGAAGCCCTC 116679836
Query 69 GACT-CA-ATTCACAGGATATAGACAAATTTATGGCTTTGG 107
Sbjct 116679837 CGCTGTAGATTCACAGGAAATAGACAAATTTATGGCTTTGG 116679877</td></tr>
<tr><td>OR13F1 rev</td><td>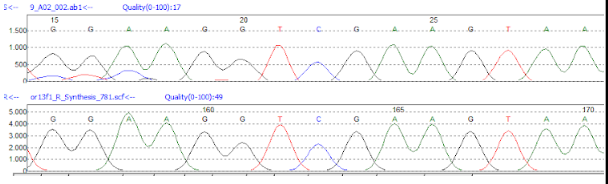
</td><td>Score 100 bits(54) · Expect 7e-19 · Identities 91/108(84%) · Gaps 6/108(5%) · Strand Plus/Minus
Features: olfactory receptor 13f1
Query 10 ACAGCGGAGGG-TTTAGGGGGAAGGA-ATATACAT-CCATAGAAC--AACTACCACCATC 64
Sbjct 116679842 ACAGCGGAGGGCTTCAGGTGCATGGAGAGAGCCATCCCATAGAACAAAACTACCACCATC 116679783
Query 65 AGGTGCGCCGGGGCACGTTGAAAAGGCTTTACTTCGACCTTCCACCGA 112
Sbjct 116679782 AGGTGGGCTGTG-CACGTTGAAAAGGCTTTACTTCGACCTTCCACTGA 116679736</td></tr>
<tr><td>OR10A5 forw</td><td>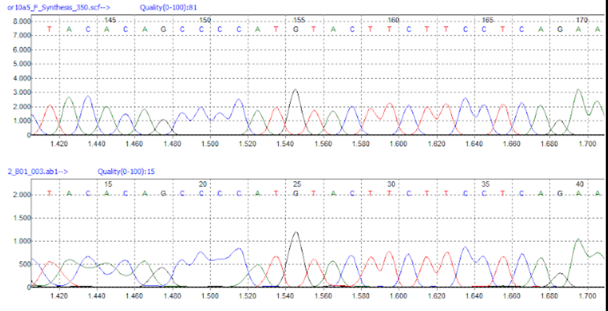
</td><td>Score 313 bits(169) · Expect 2e-82 · Identities 176/179(98%) · Gaps 2/179(1%) · Strand Plus/Plus
Features: olfactory receptor 10a5
Query 7 CATGCTACACAGCCCCATGTACTTCTTCCTCAGAAACTTATCTTTCCTGGAGATTGGCTT 66
Sbjct 6904255 CATGCTACACAGCCCCATGTACTTCTTCCTCAGAAACTTATCTTTCCTGGAGATTGGCTT 6904314
Query 67 CAAT-TAGTCATTGTGCCCAAAATGCTGGGGACCCTGCTTGCCCAGGACACAACCATCTC 125
Sbjct 6904315 CAACCTAGTCATTGTGCCCAAAATGCTGGGGACCCTGCTTGCCCAGGACACAACCATCTC 6904374
Query 126 CTTCCTTGGCTGTGCCACTCAGATGTATTTCTTCTTCTTCTTTGGGG-AGCTGAATGCT 183
Sbjct 6904375 CTTCCTTGGCTGTGCCACTCAGATGTATTTCTTCTTCTTCTTTGGGGTAGCTGAATGCT 6904433</td></tr>
<tr><td>OR10A5 rev</td><td>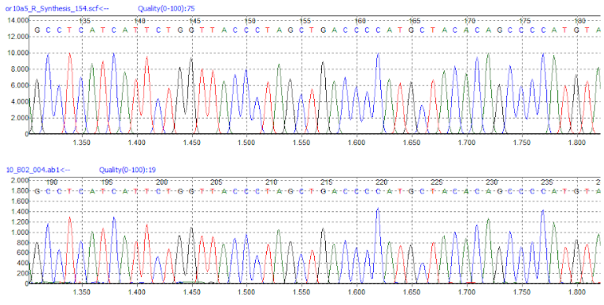
</td><td>Score 329 bits(178) · Expect 1e-87 · Identities 181/182(99%) · Gaps 1/182(0%) · Strand Plus/Minus
Features: olfactory receptor 10a5
Query 5 TCTGAGTGGCACAGCCA-GGAAGGAGATGGTTGTGTCCTGGGCAAGCAGGGTCCCCAGCA 63
Sbjct 6904397 TCTGAGTGGCACAGCCAAGGAAGGAGATGGTTGTGTCCTGGGCAAGCAGGGTCCCCAGCA 6904338
Query 64 TTTTGGGCACAATGACTAGGTTGAAGCCAATCTCCAGGAAAGATAAGTTTCTGAGGAAGA 123
Sbjct 6904337 TTTTGGGCACAATGACTAGGTTGAAGCCAATCTCCAGGAAAGATAAGTTTCTGAGGAAGA 6904278
Query 124 AGTACATGGGGCTGTGTAGCATGGGGTCAGCTAGGGTAACCAGAATGATGAGGCTGTTTC 183
Sbjct 6904277 AGTACATGGGGCTGTGTAGCATGGGGTCAGCTAGGGTAACCAGAATGATGAGGCTGTTTC 6904218
Query 184 CC 185
Sbjct 6904217 CC 6904216</td></tr>
<tr><td>OR2L5 forw</td><td>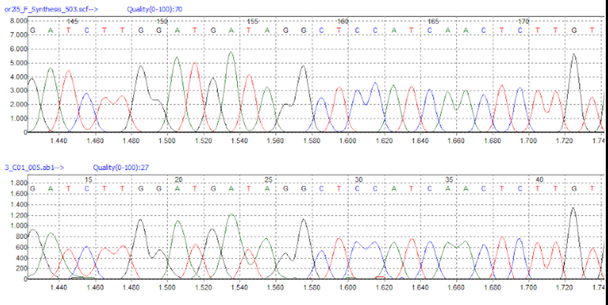
</td><td>Score 230 bits(124) · Expect 1e-57 · Identities 136/141(96%) · Gaps 3/141(2%) · Strand Plus/Plus
Features: olfactory receptor 2l5
Query 9 CAGGATCTTGGATGATAGGCTCCATCAACTCTTGTGCTCACACAGTATATGCATTCCGTA 68
Sbjct 247448433 CAGGATCTTGGATGATAGGCTCCATCAACTCTTGTGCTCACACAGTATATGCATTCCGTA 247448492
Query 69 TC-TA-ATTGCAAGTCCAGAGCCATCAATCATTTTTTCTGTGATGTCTCTAGCTATGTTG 126
Sbjct 247448493 TCCCATATTGCAAGTCCAGAGCCATCAATCATTTTTTCTGTGATGT-TCCAGCTATGTTG 247448551
Query 127 ACATTAGCCTGTACAGACACC 147
Sbjct 247448552 ACATTAGCCTGTACAGACACC 247448572</td></tr>
</table>

OR2L5 rev

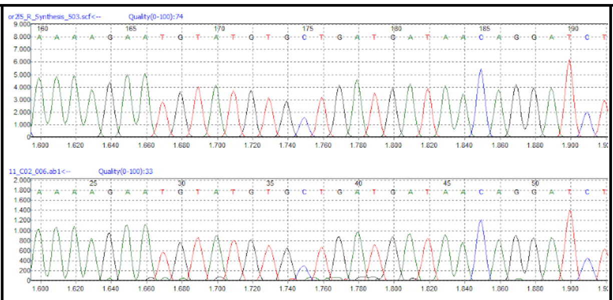

Score	Expect	Identities	Gaps	Strand
250 bits(135)	1e-63	135/135(100%)	0/135(0%)	Plus/Minus

Features: olfactory receptor 2l5

```
Query  11         AAATGATTGATGGCTCTGGACTTGCAATATGGGATACGGAATGCATATACTGTGTGAGCA  70
                  ||||||||||||||||||||||||||||||||||||||||||||||||||||||||||||
Sbjct  247448526  AAATGATTGATGGCTCTGGACTTGCAATATGGGATACGGAATGCATATACTGTGTGAGCA  247448467

Query  71         CAAGAGTTGATGGAGCCTATCATCCAAGATCCTGTTATCATCAGCACATACATTCTTTTG  130
                  ||||||||||||||||||||||||||||||||||||||||||||||||||||||||||||
Sbjct  247448466  CAAGAGTTGATGGAGCCTATCATCCAAGATCCTGTTATCATCAGCACATACATTCTTTTG  247448407

Query  131        CTCATACGGATGGGA  145
                  |||||||||||||||
Sbjct  247448406  CTCATACGGATGGGA  247448392
```

OR2B2 forw

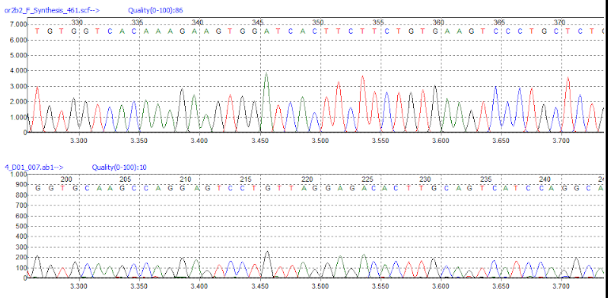

Score	Expect	Identities	Gaps	Strand
263 bits(142)	2e-67	142/142(100%)	0/142(0%)	Plus/Plus

Features: olfactory receptor 2b2

```
Query  13        CTCTGGTGCATGATAATTGAGTAATGGAGAGGCCGACAAATAGCTACAAACCTATCAAAG  72
                 ||||||||||||||||||||||||||||||||||||||||||||||||||||||||||||
Sbjct  27781554  CTCTGGTGCATGATAATTGAGTAATGGAGAGGCCGACAAATAGCTACAAACCTATCAAAG  27781613

Query  73        CACATGACGGCCAGGAGAAGACATTCTGTGGAACCCAAGGCCAGGAAAATGAAAAGCTGG  132
                 ||||||||||||||||||||||||||||||||||||||||||||||||||||||||||||
Sbjct  27781614  CACATGACGGCCAGGAGAAGACATTCTGTGGAACCCAAGGCCAGGAAAATGAAAAGCTGG  27781673

Query  133       GCCACACAGCCACCATAACTGA  154
                 ||||||||||||||||||||||
Sbjct  27781674  GCCACACAGCCACCATAACTGA  27781695
```

OR2B2 rev

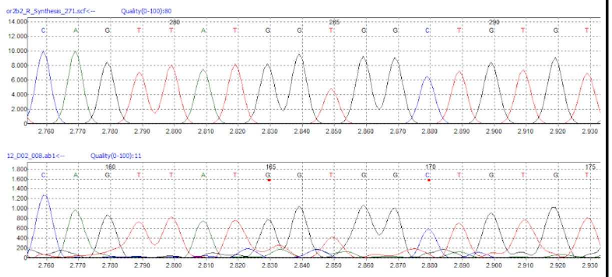

Score	Expect	Identities	Gaps	Strand
257 bits(139)	1e-65	144/146(99%)	2/146(1%)	Plus/Minus

Features: olfactory receptor 2b2

```
Query  8         CTGG-CTTGGGTT-CACAGAATGTCTTCTCCTGGCCGTCATGTGCTTTGATAGGTTTGTA  65
                 |||| |||||||| ||||||||||||||||||||||||||||||||||||||||||||||
Sbjct  27781657  CTGGCCTTGGGTTCCACAGAATGTCTTCTCCTGGCCGTCATGTGCTTTGATAGGTTTGTA  27781598

Query  66        GCTATTTGTCGGCCTCTCCATTACTCAATTATCATGCACCAGAGGCTCTGCTTCCAGTTG  125
                 ||||||||||||||||||||||||||||||||||||||||||||||||||||||||||||
Sbjct  27781597  GCTATTTGTCGGCCTCTCCATTACTCAATTATCATGCACCAGAGGCTCTGCTTCCAGTTG  27781538

Query  126       GCAGCTGCATCCTGGATTAGTGGCTT  151
                 ||||||||||||||||||||||||||
Sbjct  27781537  GCAGCTGCATCCTGGATTAGTGGCTT  27781512
```

OR9A4 forw

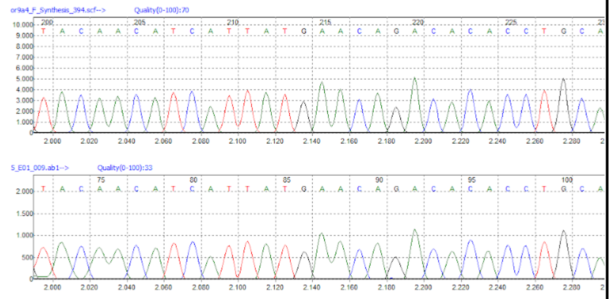

Score	Expect	Identities	Gaps	Strand
230 bits(124)	9e-58	124/124(100%)	0/124(0%)	Plus/Plus

Features: olfactory receptor 9a4

```
Query  9          ACAGAGTTCGCATTACTTGGAGCAATGGCTGTGGACCGTTATGTGGCTGTCTGTAACCCT  68
                  ||||||||||||||||||||||||||||||||||||||||||||||||||||||||||||
Sbjct  143234974  ACAGAGTTCGCATTACTTGGAGCAATGGCTGTGGACCGTTATGTGGCTGTCTGTAACCCT  143235033

Query  69         CTGAGGTACAACATCATTATGAACAGACACACCTGCAACTTTGTGGTTCTTGTGTCATGG  128
                  ||||||||||||||||||||||||||||||||||||||||||||||||||||||||||||
Sbjct  143235034  CTGAGGTACAACATCATTATGAACAGACACACCTGCAACTTTGTGGTTCTTGTGTCATGG  143235093

Query  129        GTGT  132
                  ||||
Sbjct  143235094  GTGT  143235097
```

OR9A4 rev

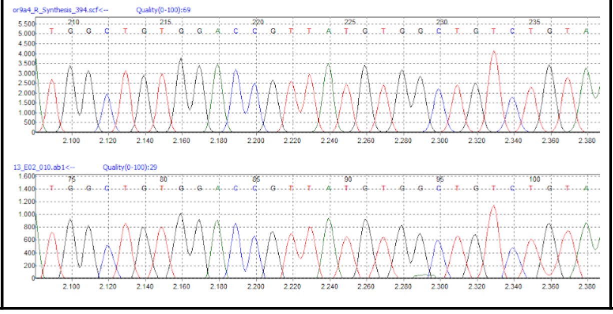

Score	Expect	Identities	Gaps	Strand
228 bits(123)	4e-57	125/126(99%)	0/126(0%)	Plus/Minus

Features: olfactory receptor 9a4

```
Query  8          TCTGTTCATAATGATGTTGTACCTCAGAGGGTTACAGACAGCCACATAACGGTCCACAGC  67
                  ||||||||||||||||||||||||||||||||||||||||||||||||||||||||||||
Sbjct  143235060  TCTGTTCATAATGATGTTGTACCTCAGAGGGTTACAGACAGCCACATAACGGTCCACAGC  143235001

Query  68         CATTGTTCCAAGTAATGCGAACTCTGTTGTCCCCACAGCAAGGTACAAGAAGAGCTGGAC  127
                  ||||| ||||||||||||||||||||||||||||||||||||||||||||||||||||||
Sbjct  143235000  CATTGCTCCAAGTAATGCGAACTCTGTTGTCCCCACAGCAAGGTACAAGAAGAGCTGGAC  143234941

Query  128        AACACA  133
                  ||||||
Sbjct  143234940  AACACA  143234935
```

OR52B6 forw

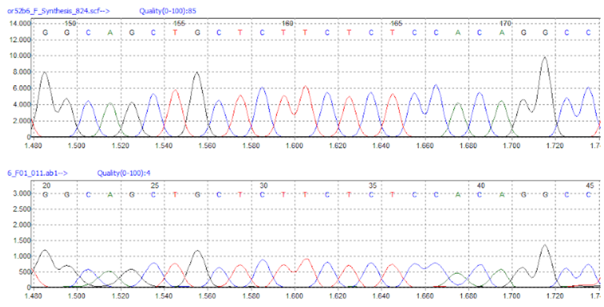

Score	Expect	Identities	Gaps	Strand
154 bits(83)	2e-34	83/83(100%)	0/83(0%)	Plus/Plus

Features: olfactory receptor 52b6

```
Query  7        TCTGGTATGGGTTGGCAGCTGCTCTTCTCTCCACAGGCCTGGACATCATGCTTATTACTG  66
                ||||||||||||||||||||||||||||||||||||||||||||||||||||||||||||
Sbjct  5640941  TCTGGTATGGGTTGGCAGCTGCTCTTCTCTCCACAGGCCTGGACATCATGCTTATTACTG  5641000

Query  67       TTTCCTACATCCACATCCTCCAA  89
                |||||||||||||||||||||||
Sbjct  5641001  TTTCCTACATCCACATCCTCCAA  5641023
```

OR52B6 rev

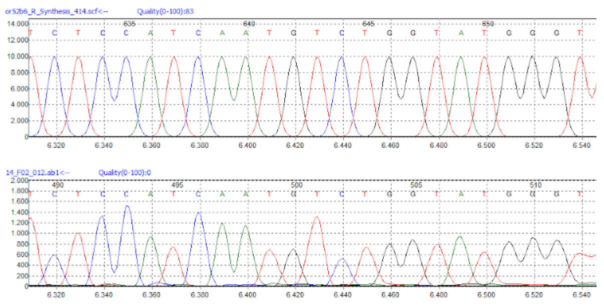

Score	Expect	Identities	Gaps	Strand
145 bits(78)	7e-32	83/85(98%)	2/85(2%)	Plus/Minus

Features: olfactory receptor 52b6

```
Query  5        TGATGT-CAGGCCTGTGGAGAGA-GAGCAGCTGCCAACCCATACCAGACATTGATGGAGA  62
                |||||| |||||||||||||||| ||||||||||||||||||||||||||||||||||||
Sbjct  5640988  TGATGTCCAGGCCTGTGGAGAGAAGAGCAGCTGCCAACCCATACCAGACATTGATGGAGA  5640929

Query  63       TATCAGAACAGGACAGATGGGCAAT  87
                |||||||||||||||||||||||||
Sbjct  5640928  TATCAGAACAGGACAGATGGGCAAT  5640904
```

OR51B5 forw

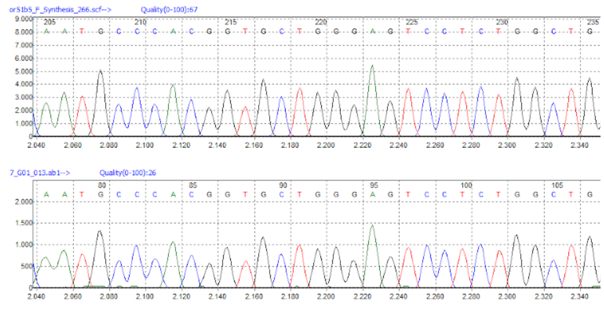

Score	Expect	Identities	Gaps	Strand
339 bits(183)	1e-90	193/197(98%)	3/197(1%)	Plus/Plus

Features: olfactory receptor 51b5
olfactory receptor 51b5

```
Query  1        GAGTGTGTGTAAGTAGGCCTGGG-AAAGCAGGCCGCACTTCCAATCTCCCTGTGATCCAG  59
                ||||||||  ||||||||||||| ||||||||||||||||||||||||||||||||||||
Sbjct  5402578  GAGTGTGT-AAAGTAGGCCTGGGAAAAGCAGGCCGCACTTCCAATCTCCCTGTGATCCAG  5402636

Query  60       CCAGAGGACTCCCAGCACCGTGGGCATTGTGGTCAGGGCCAGCCCCAGGTCTGTGGCAGC  119
                ||||||||||||||||||||||||||||||||||||||||||||||||||||||||||||
Sbjct  5402637  CCAGAGGACTCCCAGCACCGTGGGCATTGTGGTCAGGGCCAGCCCCAGGTCTGTGGCAGC  5402696

Query  120      CAGCATGGCCAGAAAGAAGTACATGGGCTCATGAAGATTGTGATCTTCCTTAATGAGAAG  179
                ||||||||||||||||||||||||||||||||||||||||||||||||||||||||||||
Sbjct  5402697  CAGCATGGCCAGAAAGAAGTACATGGGCTCATGAAGATTGTGATCTTCCTTAATGAGAAG  5402756

Query  180      AAGGAGGGGTGCCATTG  196
                |||||||| ||||||||
Sbjct  5402757  AAGGAGGG-TGCCATTG  5402772
```

OR51B5 rev

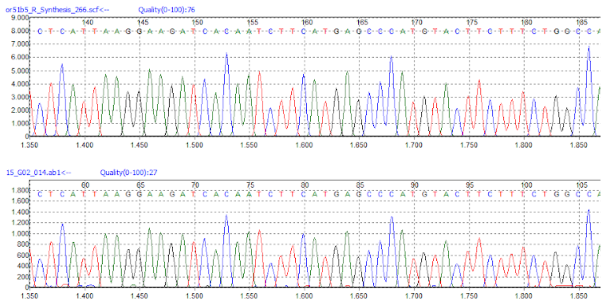

Score	Expect	Identities	Gaps	Strand
346 bits(187)	7e-93	192/194(99%)	1/194(0%)	Plus/Minus

Features: olfactory receptor 51b5
olfactory receptor 51b5

```
Query  5        CAATCTTCATGAGCCCATGTACTTCTTTCTGGCCATGCTGGCTGCCACAGACCTGGGGCT  64
                ||||||||||||||||||||||||||||||||||||||||||||||||||||||||||||
Sbjct  5402736  CAATCTTCATGAGCCCATGTACTTCTTTCTGGCCATGCTGGCTGCCACAGACCTGGGGCT  5402677

Query  65       GGCCCTGACCACAATGCCCACGGTGCTGGGAGTCCTCTGGCTGGATCACAGGGAGATTGG  124
                ||||||||||||||||||||||||||||||||||||||||||||||||||||||||||||
Sbjct  5402676  GGCCCTGACCACAATGCCCACGGTGCTGGGAGTCCTCTGGCTGGATCACAGGGAGATTGG  5402617

Query  125      AAGTGCGGCCTGCTTTTCCCAGGCCTACTTTACACACTCACTTTCCTTTCTCGAGTCTGG  184
                ||||||||||||||||||||||||||||||||||||||||||||||||||||||||||||
Sbjct  5402616  AAGTGCGGCCTGCTTTTCCCAGGCCTACTTTACACACTCACTTTCCTTTCTCGAGTCTGG  5402557

Query  185      GATTCTGCTTAGCC  198
                 ||||||||| |||
Sbjct  5402556  CATTCTGCTT-GCC  5402544
```

OR2G3 forw

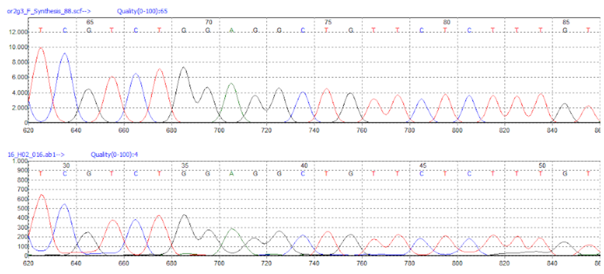

Score	Expect	Identities	Gaps	Strand
139 bits(75)	9e-31	90/97(93%)	2/97(2%)	Plus/Plus

Features: olfactory receptor 2g3

```
Query  5          ATCGTTCTA-GCTTCTCAGA-CACCCTCGTCTGGAGGCTGTTCTCTTTGTATTTGTCCTT  62
                  ||| ||||| |||||||||| |||||||||||||||||||||||||||||||||||||||
Sbjct  247042448  ATCCTTCTAGGCTTCTCAGACCACCCTCGTCTGGAGGCTGTTCTCTTTGTATTTGTCCTT  247042507

Query  63         TTCTTCTAACTAAAGACCCTTGTGGGAAACTTCACCA  99
                  |||||||| ||   |||||||||||||||||||||||
Sbjct  247042508  TTCTTCTACCTCCTGACCCTTGTGGGAAACTTCACCA  247042544
```

OR2G3 rev

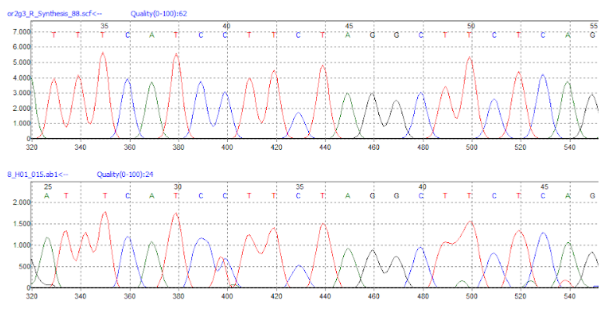


```
Score            Expect    Identities      Gaps        Strand
89.8 bits(48)    1e-15     67/75(89%)      5/75(6%)    Plus/Minus

Features:  olfactory receptor 2g3

Query  10         ATAC-AAGAG-ACAGCCT-CAGACGAGGGTGGTCTGAGAAGCCTAGAAGGATGAA-TCCA  65
                  |||| ||||| ||||||| |||||||||||||||||||||||||||||||||||| ||||
Sbjct  247042499  ATACAAAGAGAACAGCCTCCAGACGAGGGTGGTCTGAGAAGCCTAGAAGGATGAAATCCA  247042440

Query  66         CTACTGAGACTCTCA  80
                   ||  || |||||||
Sbjct  247042439  TTAGGGA-ACTCTCA  247042426
```

OR52H1 forw

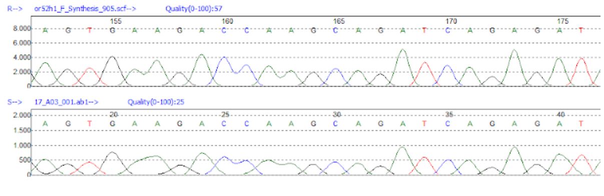


```
Score            Expect    Identities      Gaps        Strand
152 bits(82)     1e-34     84/85(99%)      0/85(0%)    Plus/Plus

Features:  olfactory receptor 52h1

Query  1        ACGTTATCTCTGATCTGCTTGGTCTTCACTCCGTAAACCATGGGGTTGAGTGCAGGTGGG  60
                || |||||||||||||||||||||||||||||||||||||||||||||||||||||||||
Sbjct  5603967  ACCTTATCTCTGATCTGCTTGGTCTTCACTCCGTAAACCATGGGGTTGAGTGCAGGTGGG  5604026

Query  61       ATAACAATGTAGAGATTGGCAAACA  85
                |||||||||||||||||||||||||
Sbjct  5604027  ATAACAATGTAGAGATTGGCAAACA  5604051
```

OR52H1 rev

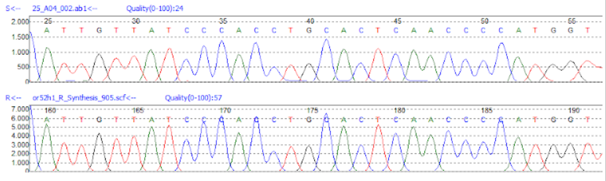


```
Score            Expect    Identities      Gaps        Strand
65.8 bits(35)    2e-08     45/49(92%)      3/49(6%)    Plus/Minus

Features:  olfactory receptor 52h1

Query  5        CCATGG-ATACGGAGTGAAGACCAAGCAGATCAGAGATAAG-T-ATACT  50
                ||||||  ||||||||||||||||||||||||||||||||| | |||||
Sbjct  5604009  CCATGGTTTACGGAGTGAAGACCAAGCAGATCAGAGATAAGGTTATACT  5603961
```

OR52K2 forw

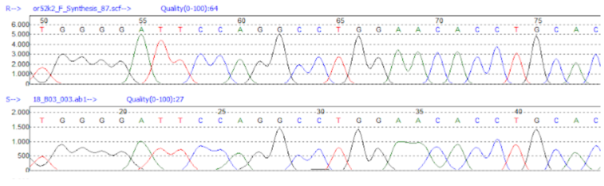


```
Score            Expect    Identities      Gaps        Strand
154 bits(83)     4e-35     83/83(100%)     0/83(0%)    Plus/Plus

Features:  olfactory receptor 52k2

Query  2        TGCCTTCTTGTTGGTGGGGATTCCAGGCCTGGAACACCTGCACATCTGGATCTCCATCCC  61
                ||||||||||||||||||||||||||||||||||||||||||||||||||||||||||||
Sbjct  4449375  TGCCTTCTTGTTGGTGGGGATTCCAGGCCTGGAACACCTGCACATCTGGATCTCCATCCC  4449434

Query  62       TTTCTGCTTAGCATATACACTGG  84
                |||||||||||||||||||||||
Sbjct  4449435  TTTCTGCTTAGCATATACACTGG  4449457
```

OR52K2 rev

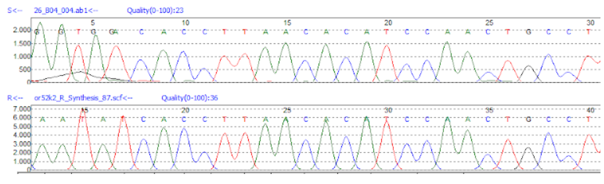


```
Score            Expect    Identities      Gaps        Strand
111 bits(60)     2e-22     63/64(98%)      1/64(1%)    Plus/Minus

Features:  olfactory receptor 52k2

Query  7        GATGTGCAGGTGTTCCAGGCCTGGA-TCCCCACCAACAAGAAGGCAGTTGGATGTGTTAA  65
                ||||||||||||||||||||||||| ||||||||||||||||||||||||||||||||||
Sbjct  4514541  GATGTGCAGGTGTTCCAGGCCTGGAATCCCCACCAACAAGAAGGCAGTTGGATGTGTTAA  4514482

Query  66       GGTG  69
                ||||
Sbjct  4514481  GGTG  4514478
```

OR2G2 forw

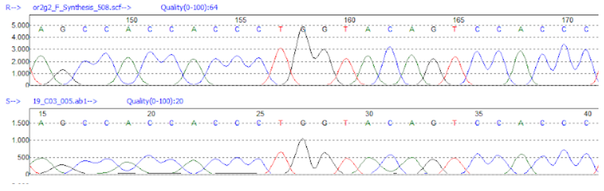


```
Score            Expect    Identities      Gaps        Strand
169 bits(91)     1e-39     98/101(97%)     1/101(0%)   Plus/Plus

Features:  olfactory receptor 2g2

Query  9          GGCATGACTCAGTGG-ATAGCCACCACCCTGGTACAGTCCACCCTCACCCCGCAGCTGCC  67
                  |||||| |||||||| |||||||||||||||||||||||||||||||||| |||||||||
Sbjct  247025652  GGCATGGCTCAGTGGAATAGCCACCACCCTGGTACAGTCCACCCTCACCCTGCAGCTGCC  247025711

Query  68         CTTCTGTGGGCATCGCCAAGTGGATCATTTCATCTGCGAGG  108
                  |||||||||||||||||||||||||||||||||||||||||
Sbjct  247025712  CTTCTGTGGGCATCGCCAAGTGGATCATTTCATCTGCGAGG  247025752
```

OR2G2 rev

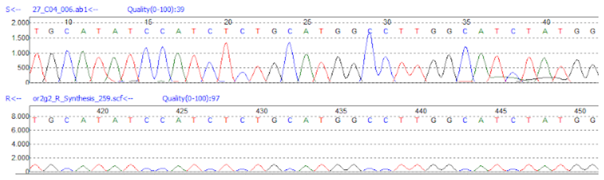


```
Score            Expect    Identities      Gaps        Strand
167 bits(90)     3e-38     92/93(99%)      0/93(0%)    Plus/Minus

Features:  olfactory receptor 2g2

Query  12         GCAGCTGCGGGGTGAGGGTGGACTGTACCAGGGTGGTGGCTATTCCACTGAGCCATGCCA  71
                  |||||||| |||||||||||||||||||||||||||||||||||||||||||||||||||
Sbjct  247588867  GCAGCTGCAGGGTGAGGGTGGACTGTACCAGGGTGGTGGCTATTCCACTGAGCCATGCCA  247588808

Query  72         TAGATGCCAAGGCCATGCAGAGATGGATATGCA  104
                  |||||||||||||||||||||||||||||||||
Sbjct  247588807  TAGATGCCAAGGCCATGCAGAGATGGATATGCA  247588775
```

OR13D1 forw

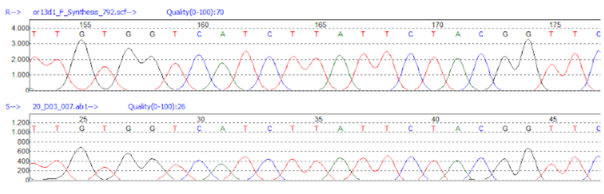


```
Score            Expect    Identities      Gaps        Strand
241 bits(130)    8e-61     136/139(98%)    0/139(0%)   Plus/Plus

Features:  olfactory receptor 13d1

Query  7          CTGATTAGCGCACTTGATTGTGGTCATCTTATTCTACGGTTCAGCCCTTTTTATGTACAT  66
                  ||| | |||||||| |||||||||||||||||||||||||||||||||||||||||||||
Sbjct  116870015  CTGTTCAGCGCACTCGATTGTGGTCATCTTATTCTACGGTTCAGCCCTTTTTATGTACAT  116870074

Query  67         GAAACCCAAGTCAAAGAACACTAATACATCTGATGAGATTATTGGGCTGTCTTATGGAGT  126
                  ||||||||||||||||||||||||||||||||||||||||||||||||||||||||||||
Sbjct  116870075  GAAACCCAAGTCAAAGAACACTAATACATCTGATGAGATTATTGGGCTGTCTTATGGAGT  116870134

Query  127        GGTAAGCCCAATGTTAAAT  145
                  |||||||||||||||||||
Sbjct  116870135  GGTAAGCCCAATGTTAAAT  116870153
```

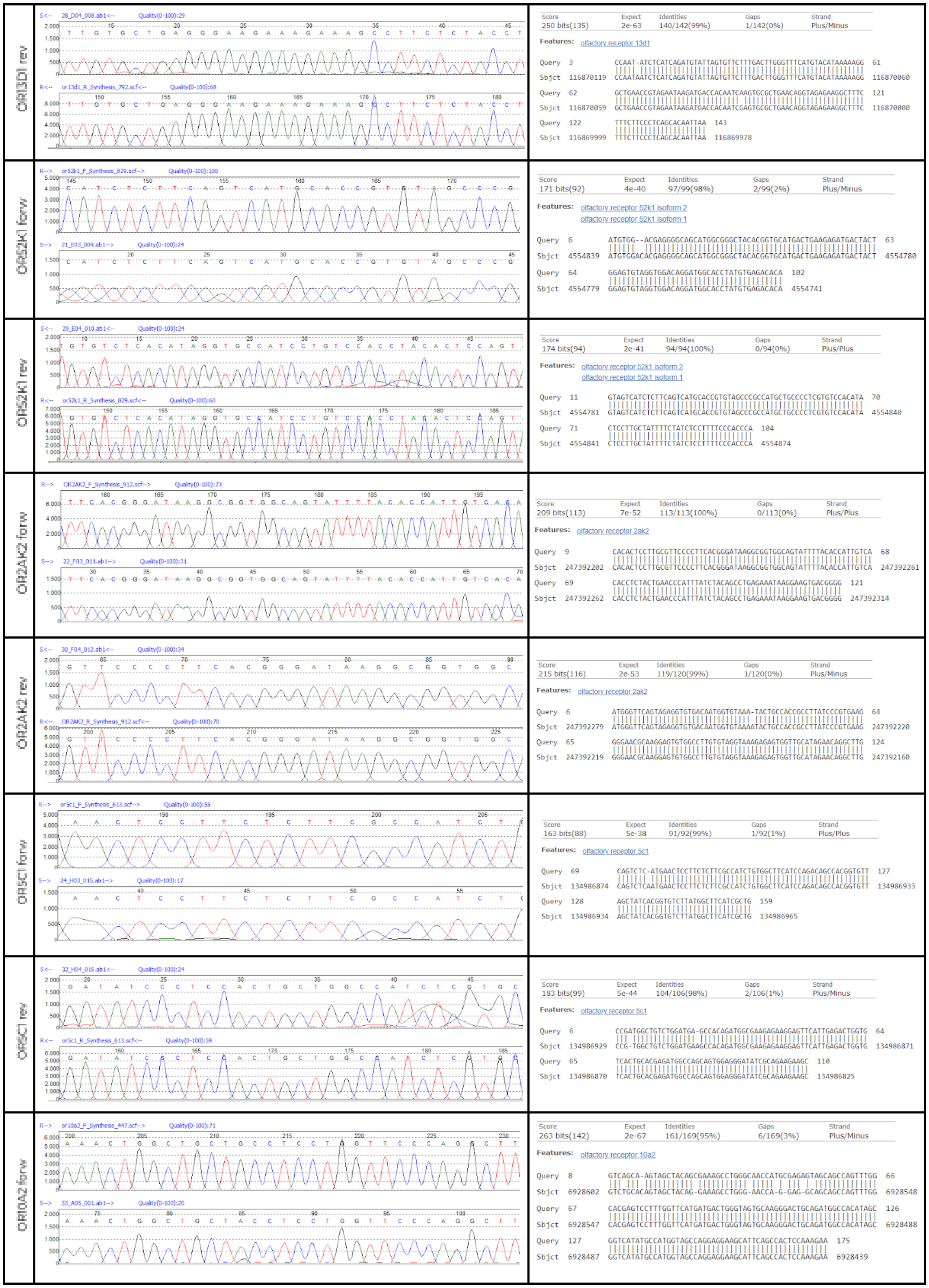

OR13D1 rev
Score 250 bits(135) Expect 2e-63 Identities 140/142(99%) Gaps 1/142(0%) Strand Plus/Minus
Features: olfactory receptor 13d1
OR52K1 forw
Score 171 bits(92) Expect 4e-40 Identities 97/99(98%) Gaps 2/99(2%) Strand Plus/Minus
Features: olfactory receptor 52k1 isoform 2
olfactory receptor 52k1 isoform 1
OR52K1 rev
Score 174 bits(94) Expect 2e-41 Identities 94/94(100%) Gaps 0/94(0%) Strand Plus/Plus
Features: olfactory receptor 52k1 isoform 2
olfactory receptor 52k1 isoform 1
OR2AK2 forw
Score 209 bits(113) Expect 7e-52 Identities 113/113(100%) Gaps 0/113(0%) Strand Plus/Plus
Features: olfactory receptor 2ak2
OR2AK2 rev
Score 215 bits(116) Expect 2e-53 Identities 119/120(99%) Gaps 1/120(0%) Strand Plus/Minus
Features: olfactory receptor 2ak2
OR5C1 forw
Score 163 bits(88) Expect 5e-38 Identities 91/92(99%) Gaps 1/92(1%) Strand Plus/Plus
Features: olfactory receptor 5c1
OR5C1 rev
Score 183 bits(99) Expect 5e-44 Identities 104/106(98%) Gaps 2/106(1%) Strand Plus/Minus
Features: olfactory receptor 5c1
OR10A2 forw
Score 263 bits(142) Expect 2e-67 Identities 161/169(95%) Gaps 6/169(3%) Strand Plus/Minus
Features: olfactory receptor 10a2

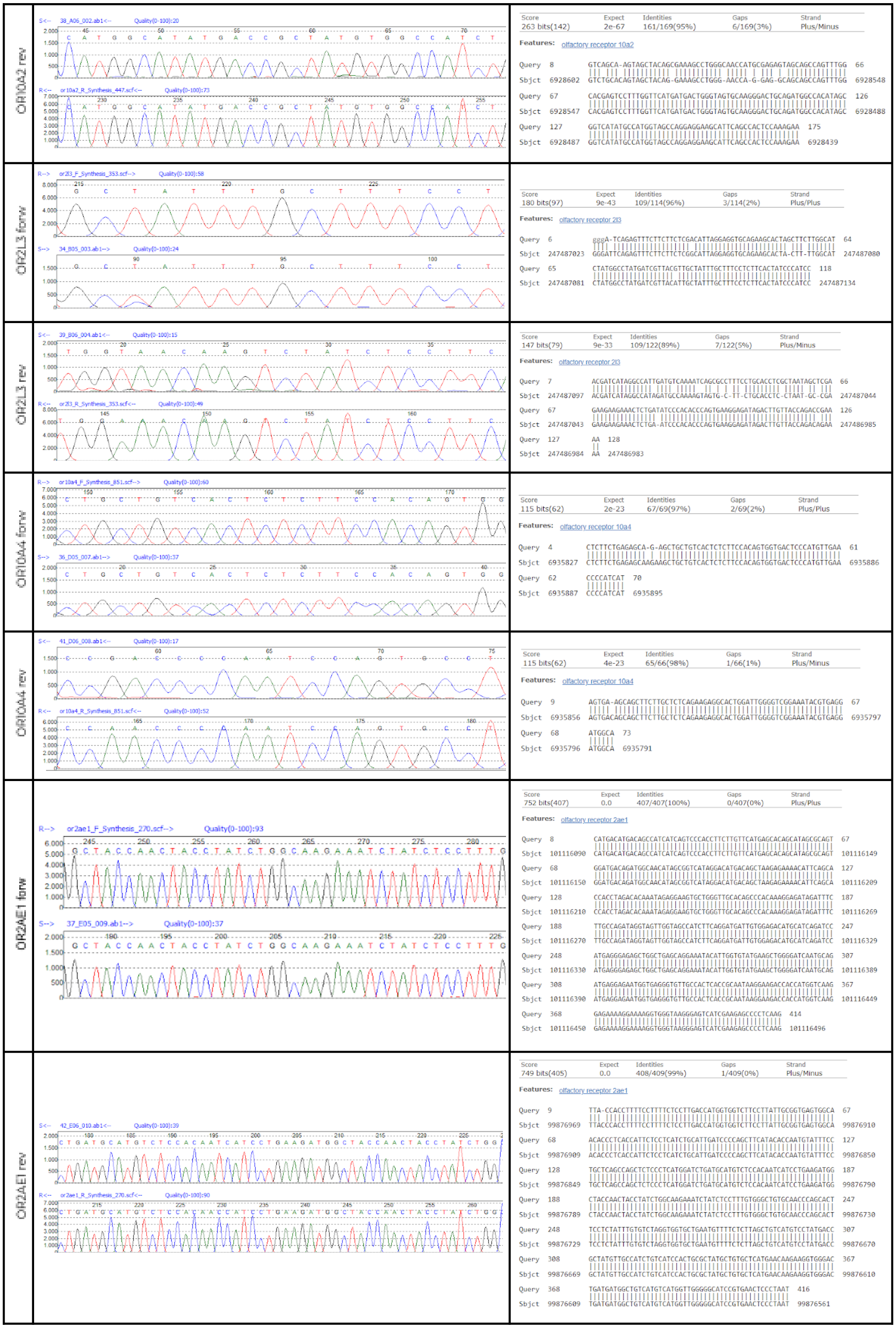

OR10A2 rev
Score 263 bits(142) Expect 2e-67 Identities 161/169(95%) Gaps 6/169(3%) Strand Plus/Minus
Features: olfactory receptor 10a2
OR2L13 forw
Score 180 bits(97) Expect 9e-43 Identities 109/114(96%) Gaps 3/114(2%) Strand Plus/Plus
Features: olfactory receptor 2l13
OR2L13 rev
Score 147 bits(79) Expect 9e-33 Identities 109/122(89%) Gaps 7/122(5%) Strand Plus/Minus
Features: olfactory receptor 2l13
OR10A4 forw
Score 115 bits(62) Expect 2e-23 Identities 67/69(97%) Gaps 2/69(2%) Strand Plus/Plus
Features: olfactory receptor 10a4
OR10A4 rev
Score 115 bits(62) Expect 4e-23 Identities 65/66(98%) Gaps 1/66(1%) Strand Plus/Minus
Features: olfactory receptor 10a4
OR2AE1 forw
Score 752 bits(407) Expect 0.0 Identities 407/407(100%) Gaps 0/407(0%) Strand Plus/Plus
Features: olfactory receptor 2ae1
OR2AE1 rev
Score 749 bits(405) Expect 0.0 Identities 408/409(99%) Gaps 1/409(0%) Strand Plus/Minus
Features: olfactory receptor 2ae1

OR1L6 forw	Unmatched (Em repetição)	
OR1L6 rev	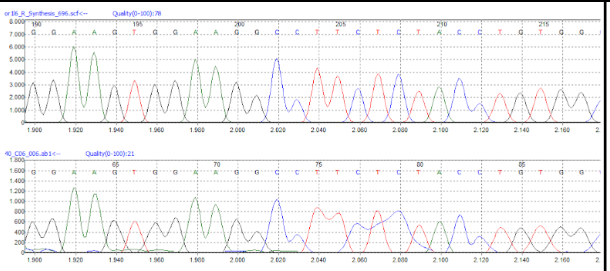	Score 167 bits(90) · Expect 6e-39 · Identities 92/93(99%) · Gaps 0/93(0%) · Strand Plus/Minus<br>Features: olfactory receptor 1l6<br>Query 15 TACTGCAGTGAGGTGGGAGCCACAGGTAGAGAAGGCCTTCCACTTCCTGGCTGCAGAGGG 74<br>Sbjct 122750594 TACTGCAGTGAGGTGGGAGCCACAGGTAGAGAAGGCCTTCCACTTCCCGGCTGCAGAGGG 122750535<br>Query 75 GATTCTGAGCACAGTGACCATGATTCGCAGGTA 107<br>Sbjct 122750534 GATTCTGAGCACAGTGACCATGATTCGCAGGTA 122750502
OR4X2 forw	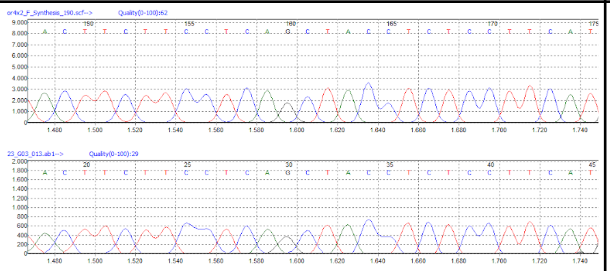	Score 145 bits(78) · Expect 2e-32 · Identities 83/85(98%) · Gaps 2/85(2%) · Strand Plus/Plus<br>Features: olfactory receptor 4x2<br>Query 4 CTTGGTT--CCCATGTACTTCTTCCTCAGCTACCTCTCCTTCATGGAGATCTGCTACTCC 61<br>Sbjct 48414732 CTTGGTTCCCCCATGTACTTCTTCCTCAGCTACCTCTCCTTCATGGAGATCTGCTACTCC 48414791<br>Query 62 TCCGCTACAGCCCCCAAACTCATCT 86<br>Sbjct 48414792 TCCGCTACAGCCCCCAAACTCATCT 48414816
OR4X2 rev	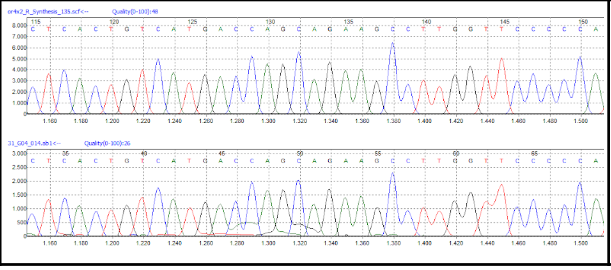	Score 174 bits(94) · Expect 2e-41 · Identities 97/98(99%) · Gaps 1/98(1%) · Strand Plus/Minus<br>Features: olfactory receptor 4x2<br>Query 6 GGAGATAGCAGATCTCCATGAAGGAGAGGTAGCTGAGGAAGAAGTACATGGGGAACCAA 65<br>Sbjct 48414791 GGAG-TAGCAGATCTCCATGAAGGAGAGGTAGCTGAGGAAGAAGTACATGGGGAACCAA 48414733<br>Query 66 GGCTTCTGCTGGTCATGACAGTGAGCACAATGAGGAAA 103<br>Sbjct 48414732 GGCTTCTGCTGGTCATGACAGTGAGCACAATGAGGAAA 48414695

Figure S3. Confirmation of specificity of ORs PCRs. Since ORs have 40-90% homology with each other, all OR PCRs were double checked with forward and reverse sequencing. A healthy donor genomic DNA was used to amplify all OR PCRs. The first column shows the electropherogram aligned to the corresponding reference sequence in Mutation Surveyor Software (Soft Genetics), following, the sequence was aligned to the human genome with the BLAST tool (NCBI). The majority of sequences aligned over 95% with the corresponding targeted OR in at least forward or reverse sequencing, with exception of OR13F1, which showed 92% of alignment. As expected, in some BLAST analysis, the corresponding OR was not the only target listed, however, it was the target with highest score and longer and highest identity (bp, %).

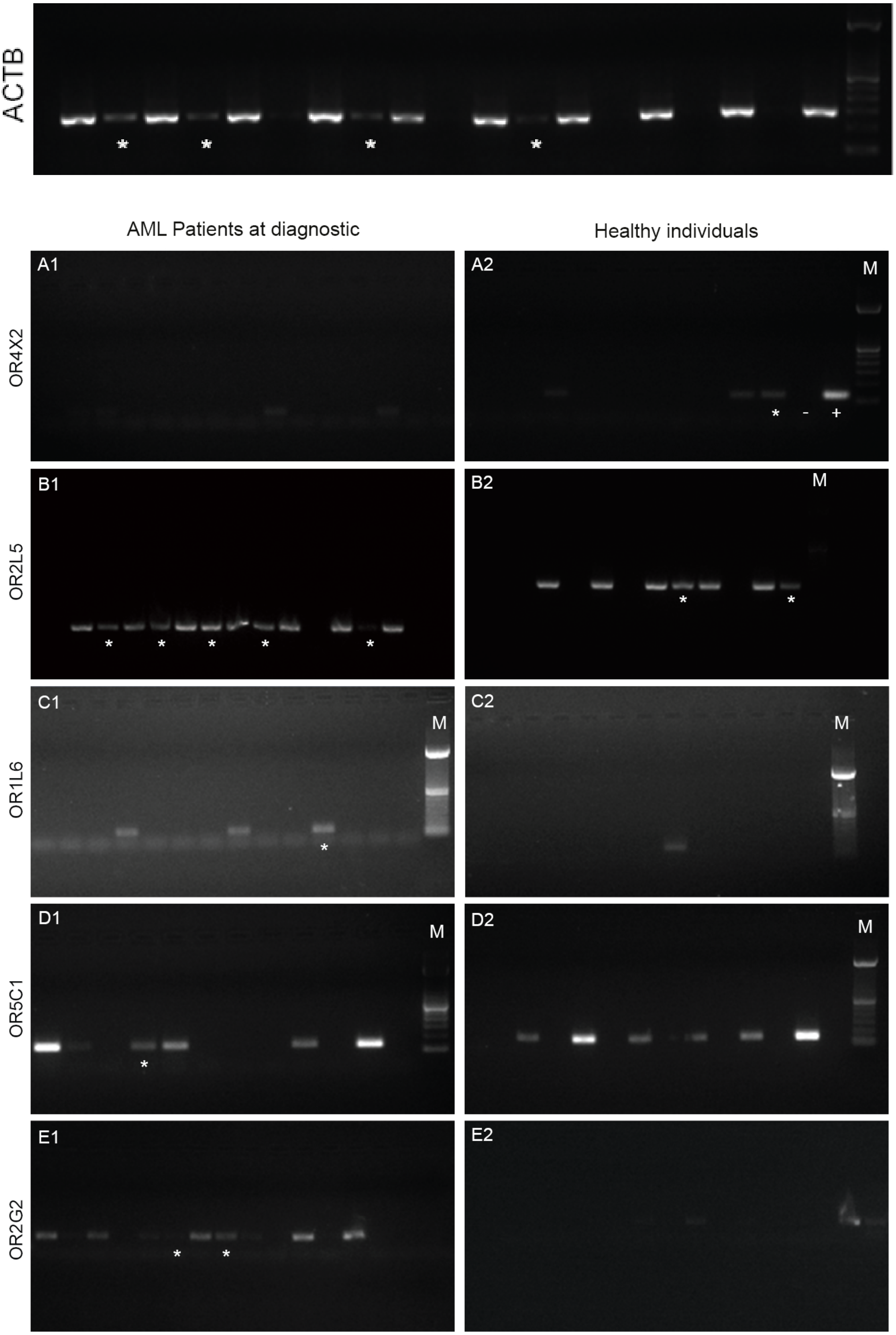
ACTB
AML Patients at diagnostic
Healthy individuals
A1
A2
M
OR4X2
*
-
+
B1
B2
M
OR2L5
C1
C2
M
OR1L6
D1
D2
M
OR5C1
E1
E2
OR2G2

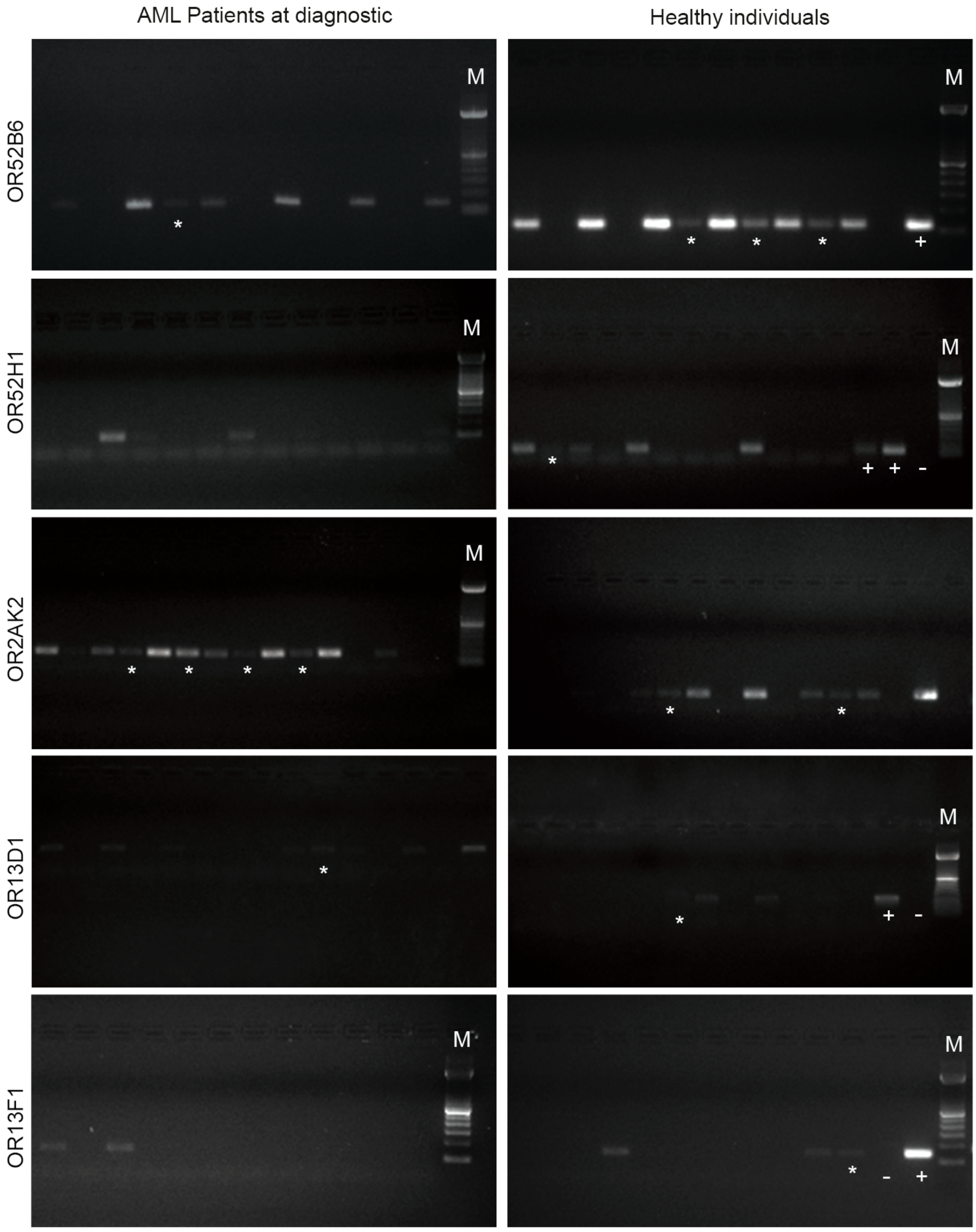

Figure S4. Expression of ORs in AML patients or healthy donors samples.

PCR products subjected to electrophoresis in agarose gel 1.5% stained with Ethidium Bromide. PCR were performed in patient or donor cDNA

synthesized with and without RT enzyme, disposed in this sequence in gel for ACTB and ORs PCR. All samples were pre-selected with PCR for ACTB (295 bp), each sample with amplification in cDNA synthesized without RT enzyme (marked by an asterisk), were removed from our cohort. However, despite this selection, some cDNA without RT were positive for one or more OR PCR and were also removed from our cohort (marked with asterisk). PCR products for OR4X2 (131 bp, A), found not expressed in AML or donor samples; OR2L5 (180 bp, B); OR1L6 (142 bp, C); OR5C1 (140 bp; D); OR2G2 (136bp; E); OR52B6 (120 bp; F); OR52H1 (114 bp; G); OR2AK2 (155 bp; H); OR13D1 (171 bp; I); OR13F1 (150 bp; J). All PCRs were confirmed in at least two independent experiments.

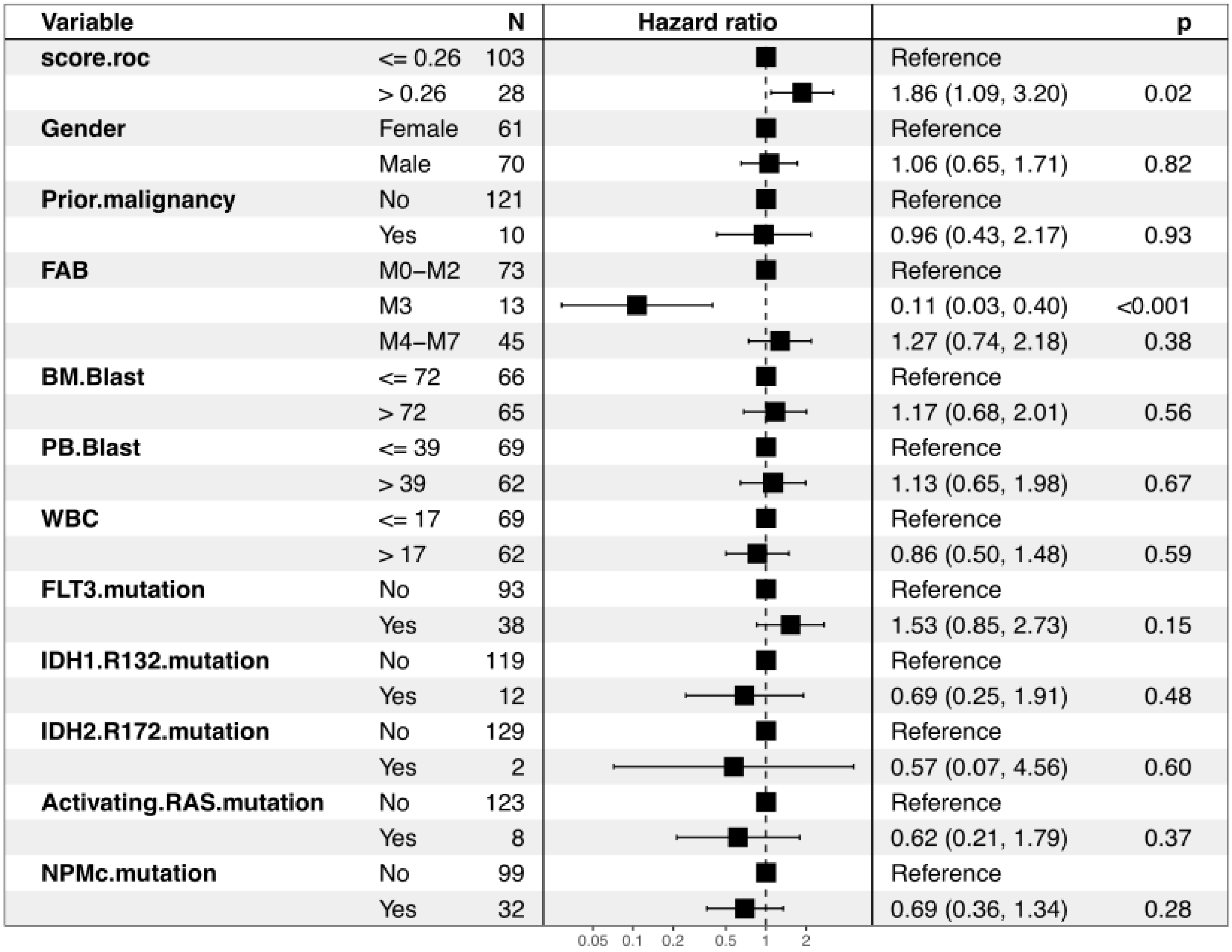

Variable		N	Hazard ratio		p
score.roc	<= 0.26	103		Reference	
	> 0.26	28		1.86 (1.09, 3.20)	0.02
Gender	Female	61		Reference	
	Male	70		1.06 (0.65, 1.71)	0.82
Prior.malignancy	No	121		Reference	
	Yes	10		0.96 (0.43, 2.17)	0.93
FAB	M0–M2	73		Reference	
	M3	13		0.11 (0.03, 0.40)	<0.001
	M4–M7	45		1.27 (0.74, 2.18)	0.38
BM.Blast	<= 72	66		Reference	
	> 72	65		1.17 (0.68, 2.01)	0.56
PB.Blast	<= 39	69		Reference	
	> 39	62		1.13 (0.65, 1.98)	0.67
WBC	<= 17	69		Reference	
	> 17	62		0.86 (0.50, 1.48)	0.59
FLT3.mutation	No	93		Reference	
	Yes	38		1.53 (0.85, 2.73)	0.15
IDH1.R132.mutation	No	119		Reference	
	Yes	12		0.69 (0.25, 1.91)	0.48
IDH2.R172.mutation	No	129		Reference	
	Yes	2		0.57 (0.07, 4.56)	0.60
Activating.RAS.mutation	No	123		Reference	
	Yes	8		0.62 (0.21, 1.79)	0.37
NPMc.mutation	No	99		Reference	
	Yes	32		0.69 (0.36, 1.34)	0.28

Figure S5. Multivariate Cox survival analysis of the OR expression signature in AML samples from TCGA.

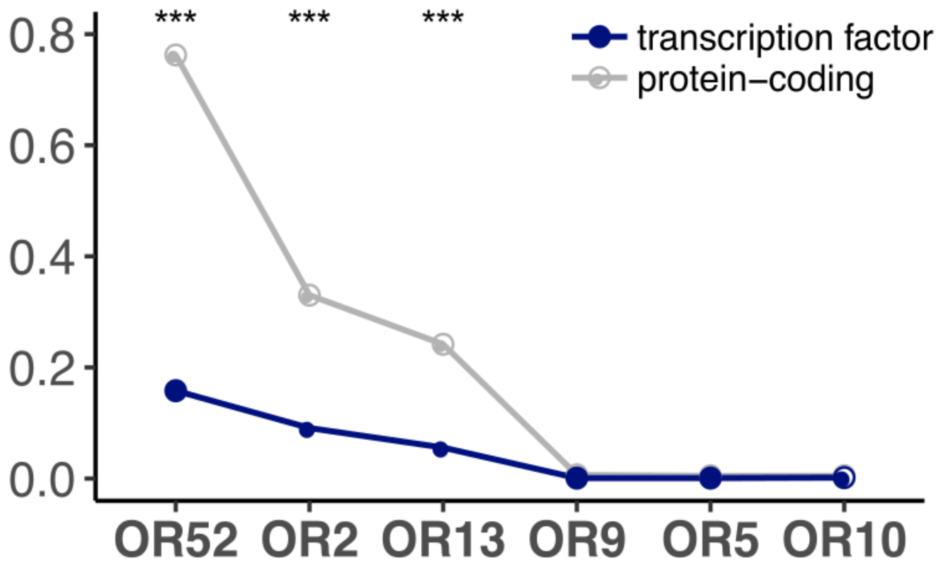

Figure S6. Enrichment of transcription factors coexpressed with ORs from subfamilies OR2, OR13 and OR52.
